# Surgical hand preparation in an equine hospital: Comparison of general practice with a standardised protocol and characterisation of the methicillin-resistant *Staphylococcus aureus* recovered

**DOI:** 10.1371/journal.pone.0242961

**Published:** 2020-12-22

**Authors:** Tina Rocktäschel, Katharina Renner-Martin, Christiane Cuny, Walter Brehm, Uwe Truyen, Stephanie Speck

**Affiliations:** 1 Institute of Animal Hygiene and Veterinary Public Health, Faculty of Veterinary Medicine, University of Leipzig, Leipzig, Germany; 2 Institute of Mathematics, Department of Integrative Biology and Biodiversity Research, University of Natural Resources and Life Sciences, Vienna, Austria; 3 Robert Koch Institute, National Reference Centre for Staphylococci and Enterococci, Wernigerode, Germany; 4 Department for Horses, Faculty of Veterinary Medicine, University of Leipzig, Leipzig, Germany; University of Camerino, ITALY

## Abstract

Presurgical hand asepsis is part of the daily routine in veterinary medicine. Nevertheless, basic knowledge seems to be low, even among specialised veterinary surgeons. The major objectives of our study were to assess current habits for presurgical hand preparation (phase 1) among personnel in a veterinary hospital and their effectiveness in reducing bacteria from hands in comparison to a standardised protocol (phase 2). Assessment of individual habits focused on time for hand washing and disinfection, the amount of disinfectant used, and the usage of brushes. The standardised protocol defined hand washing for 1 min with liquid neutral soap without brushing and disinfection for 3 min. All participants (2 surgeons, 8 clinic members, 32 students) used Sterillium^®^. Total bacterial counts were determined before and after hand washing, after disinfection, and after surgery. Hands were immersed in 100 ml sterile sampling fluid for 1 min and samples were inoculated onto Columbia sheep blood agar using the spread-plate method. Bacterial colonies were manually counted. Glove perforation test was carried out at the end of the surgical procedure. Differences in the reduction of relative bacterial numbers between current habits and the standardised protocol were investigated using Mann-Whitney-Test. The relative increase in bacterial numbers as a function of operation time (≤60 min, >60 min) and glove perforation as well as the interaction of both was investigated by using ANOVA. Forty-six and 41 preparations were carried out during phase 1 and phase 2, respectively. Individual habits differed distinctly with regard to time (up to 8 min) and amount of disinfectant (up to 48 ml) used both between participants and between various applications of a respective participant. Comparison of current habits and the standardised protocol revealed that the duration of hand washing had no significant effect on reducing bacteria. Contrary, the reduction in bacterial numbers after disinfection by the standardised protocol was significantly higher (p<0.001) compared to routine every-day practice. With regard to disinfection efficacy, the standardised protocol completely eliminated individual effects. The mean reduction in phase 1 was 90.72% (LR = 3.23; right hand) and 89.97% (LR = 3.28; left hand) compared to 98.85% (LR = 3.29; right hand) and 98.92% (LR = 3.47; left hand) in phase 2. Eight participants (19%) carried MRSA (*spa* type t011, CC398) which is well established as a nosocomial pathogen in veterinary clinics. The isolates could further be assigned to a subpopulation which is particularly associated with equine clinics (mainly t011, ST398, gentamicin-resistant). Glove perforation occurred in 54% (surgeons) and 17% (assistants) of gloves, respectively, with a higher number in long-term invasive procedures. Overall, bacterial numbers on hands mainly increased over time, especially when glove perforation occurred. This was most distinct for glove perforations on the left hand and with longer operating times. Our results demonstrate that standardised protocols highly improve the efficacy of hand asepsis measures. Hence, guiding standardised protocols should be prerequisite to ensure state-of-the-art techniques which is essential for a successful infection control intervention.

## Introduction

In human medicine, surgical hand asepsis is an established measure to prevent surgical site infections (SSI) and the same is likely true in veterinary medicine [1, 2]. SSI are most commonly caused by microbes of the transient skin flora [3]. However, even though presurgical hand asepsis is the most effective preventive measure for SSI and part of the daily routine, basic knowledge seems to be low, even among specialised veterinary surgeons [4]. Moreover, compliance to adhere permanently to an established standard protocol is rather missing. According to a survey amongst ACVS and ECVS surgical specialists, 66% did not adhere to state-of-the-art protocols using hydro-alcoholic solutions [4]. SSI rates up to 52.6% were reported for equine surgical procedures but it has also been assumed that approximately 35% of SSI are not documented in the medical record system [5–7]. How many infections are diagnosed anyplace after discharge of the patients remains unknown [4]. SSI are associated with prolonged hospitalisation, higher treatment costs, and may also lead to higher morbidity and mortality rates [8–11]. Methicillin-resistant *Staphylococcus aureus* (MRSA) could increase the risk for the development of SSI [11–13]. More than 6% of SSI have been associated with MRSA in an equine veterinary hospital in the UK whereas a large German study found MRSA to be associated with 9.4% of wound infections in horses [13, 14]. In recent years, MRSA of the clonal complex (CC) 398 seem to have been widely distributed in equine hospitals in Europe [15]. So far, MRSA screenings are not routinely performed in German veterinary hospitals, although 2.2–9.3% of admitted horses are carriers and could therefore spread resistant strains to other patients, veterinary staff and the environment [8, 16, 17]. Moreover, 20% of veterinarians and staff in equine clinics have been reported to be nasally colonised by MRSA [18]. Unintentional transfer of microorganisms to patients during surgery and treatment may therefore also occur. Wearing surgical gloves constitutes a barrier against transmission of bacteria from hands to surgical wounds but an incidence of glove punctures in large animal surgery of up to 66% highlights the necessity of an adequate and reliable hand disinfection procedure [19, 20]. Compared to human medicine, there are only few studies that focused on the efficacy of disinfectants for presurgical hand asepsis in veterinary medicine; some of these included different hand disinfection products and techniques, determined bacterial counts and species [21–25]. Mainly alcohol-based gels, alcohol-based hand rubs with additional active ingredients (Avagard^®^, Sterillium^®^), hand scrubs and soaps containing chlorhexidine and PVP-iodine were compared for reduction of aerobic bacterial counts. In some of these studies, practical guidance on the steps of surgical hand preparation was given according to WHO guidelines [23–26]. The amount of product used and the application times were chosen according to the manufacturers’ recommendations [23–25]. None of these studies evaluated the efficacy of a disinfectant using different application protocols.

The objectives of this study were 1) to assess current habits for presurgical hand preparation among personnel in an equine hospital and veterinarians-in-training, and 2) to compare the efficacy of hand asepsis done as routine every-day practice (phase 1) versus a standardised protocol (phase 2) in reducing bacteria from hands. The hypothesis was that a standardised protocol would be more efficient in reducing bacterial numbers. We further determined the prevalence of MRSA on hands before and after hand washing, after disinfection, and after surgery. Further, MRSA isolates were characterised with regard to the subpopulation of MRSA CC398 associated with equine hospitals.

## Material and methods

The study was conducted at the department for horses of a veterinary teaching hospital. It was implemented in the daily routine at the clinic. Forty-two participants volunteered in the study: two equine surgeons (surgeon A and B), eight staff members, and 32 students. The study was performed as a subproject of a large perennial survey about “Zoonotic transmission of LA-MRSA to humans exposed to livestock” (permission no. 47/09, Ethics Committee of Otto von Guericke University Magdeburg, 09/04/2009). Human sample collection was permitted for skin and mucosa of the nasal vestibulum. Specifications included the information of each participant about the methodology and aims of the study prior to participation, informed consent of each participant, and personal communication of results. Orally informed consent was obtained from all volunteers prior to participation. Bacterial numbers on hands were determined (1) prior to hand washing, (2) after washing and drying hands with a sterile towel, (3) after surgical hand disinfection, and (4) after surgery. For each surgical intervention the total time (min) of surgery and the type of surgical procedure (minimally invasive, invasive) were recorded. For analyses, surgeries were categorised in ≤60 min and >60 min. All samples collected were assigned to numbers, and data were analysed in the laboratory anonymously. Prior to each presurgical preparation, detailed information was queried using a standardised in-house questionnaire with main focus on the participants’ previous activities on that day (S1 Table). All participants used Sterillium^®^ (Bode Chemie GmbH, Hamburg, Germany) an alcohol-based hand rub containing Propan-2-ol (45 g/100 g), Propan-1-ol (30 g/100 g), and mecetronium etilsulfate (0.2 g/100 g) which belongs to the quarternary ammonium compounds. The study consisted of two parts (phase 1 and 2) which are described in detail below.

### Glove perforation test

The majority of participants wore sterile non-powdered latex gloves (Vasco OP Sensitive, 0.21 mm on the palm and 0.175 mm at the cuff; B. Braun Melsungen AG). Sterile latex-free polyisoprene gloves (Vasco OP Free, 0.21 mm on the palm and 0.205 mm at the cuff; B. Braun Melsungen AG) were used by surgeon B due to a latex allergy. According to the manufacturer, the used gloves revealed an acceptable quality level (AQL) of 0.65 which corresponds to category III [27]. Double-gloving was not practiced at any time. Immediately after termination of the surgical procedure, gloves were taken off and visually tested for the presence of lesions by a modified water leak test (WLT) [20]. Briefly, gloves were filled with coloured water, tightly closed and gently pressed. Any leak and the location of perforations were recorded for each glove. Occurrence of glove perforation was determined with regard to the type of surgery and operation time (≤60 min, >60 min). In addition, participants were asked for notice of glove perforation.

### Sample collection

Samples were collected from both hands of each participant. This corresponded to the assumption that glove punctures, which often occur only on one hand (most frequently the nondominant hand) [20], would influence bacterial counts on hands after surgery. Each hand was immersed in 100 ml sterile phosphate buffered saline (PBS, pH 7.2) filled into polypropylene bags (Sarstedt, Nümbrecht, Germany) while holding the bag tightly around the participant´s wrist as described by Traub-Dargatz et al. [21]. Prior to sample collection at the hospital, we compared PBS and neutralizer EIII (recommended by the German Association for Applied Hygiene, VAH) for their suitability as sampling fluid. EIII was composed of tryptic soy broth (TSB) with 0.3% lecithin, 0.1% L-histidine, 0.5% sodium thiosulfate, and 3% Tween 80. Both fluids were tested by twelve volunteers each after presurgical hand preparation using the standardised protocol (see study phase 2). Samples were cultured and bacterial numbers were enumerated as described below. As expected, higher bacterial counts were obtained using EIII compared to PBS. Results obtained with both sampling fluids can be taken from S2 Table. In contrast to PBS, neutralizer EIII left a sticky feel on hands, which was judged unsuitable with regard to gloving. Another step of hand washing would have influenced residual effects of the disinfectant as well as bacterial numbers on hands. Hence, we decided to use PBS without any neutralising ingredient. During the sampling process, PBS was thoroughly dispersed all-over the surfaces of each hand for 1 min. Thereafter, bags were tightly closed, placed on crushed ice and samples were further processed within 15 min after collection.

### Sample processing

Each sample obtained before and after washing, and after surgery was diluted by two serial tenfold dilutions in PBS. Samples taken after disinfection were used undiluted and in a dilution of 1:10. A 100 μl-aliquot of each undiluted and serially diluted sample was inoculated onto Columbia agar plates (5% sheep blood; OXOID Thermo Fisher Scientific, Wesel, Germany) in duplicates using sterile spreaders. Plates were incubated at 37°C for 24 h and subsequently examined for colonies suggestive of *Staphylococcus* (*S*.) *aureus* and other bacteria that might be associated with wound infection in horses, like *S*. *pseudintermedius*, *S*. *lugdunensis*, streptococci, and coliforms [28–32]. Subcultures were taken and the original plates kept under CO_2_-atmosphere (AnaeroGen, OXOID) in a 2.5-liter anaerobic jar for another 24 h. After 48 h of incubation, bacterial colonies were manually counted by the lead author and mean colony-forming units (cfu)/ml were calculated. Isolates were identified by MALDI TOF elsewhere (Diagnosticum, Neukirchen, Germany).

### Typing of MRSA

*S*. *aureus* were subjected to antimicrobial susceptibility testing and were further characterised by *spa* typing as described [33]. Related *spa* types (cost values ≤4) were grouped into *spa*-clonal clusters (*spa* CCs) using the BURP algorithm (Ridom StaphType software version 2.2.1, Ridom GmbH, Würzburg, Germany). *Spa*-CCs were allocated to MLST CCs through the *spa*-Server database (www.spaserver.ridom.de) and the database of the German National Reference Centre for staphylococci and enterococci. Discrimination of the equine hospital associated subpopulation from the general animal adapted population of CC398 (LA-MRSA C398) was performed by PCR using generate primers as previously described [18]. Antimicrobial susceptibility testing was performed by using the broth microdilution method according to DIN 58940 and applying EUCAST breakpoints for interpretation of the results (www.eucast.org). The following antibiotics were tested: penicillin, oxacillin, cefoxitin, fosfomycin, gentamicin, linezolid, erythromycin, clindamycin, tetracycline, tigecycline, vancomycin, teicoplanin, ciprofloxacin, mupirocin, moxifloxacin, daptomycin, fusidic acid-sodium, rifampicin and trimethoprim/sulfamethoxazole.

### Phase 1—routine practice protocol (status quo)

For status quo-analysis, surgeons and assistants did prepare in their respective daily routine. Students were guided in presurgical hand asepsis by the respective surgeon on the spot. Prior to presurgical preparation, the handles of the disinfectant dispensers were pulled down two to three times to deflate the small tube inside the dispenser. Each participant started with hand washing using a liquid, neutral, non-antiseptic soap (Lifosan^®^ soft, B. Braun Melsungen AG). Presurgical preparation of each participant was observed and recorded by the lead author in detail with focus on the hand washing technique (i.e. usage of nail cleaners, sponges and brushes), the frequency of soaping and rinsing, and the time taken for soaping and rinsing. The amount of disinfectant used by everyone was deduced from the number of hubs taken from the dispenser. The volume (ml)/hub was measured in a series of preceding tests by pressing the handle of the dispenser all the way down. In addition, the distribution of disinfectant and the rubbing technique were noted, and the time was measured for the duration of the rub-in procedure. Hands were gloved after complete evaporation of Sterillium^®^. Each surgeon prepared for ten surgical interventions in study phase 1. Three staff members participated in more than one surgery but students participated only once. The timely distance between presurgical preparations was at least one day, with the exception of surgeon B who once had two surgeries the same day. The number of presurgical preparations conducted in phase 1 is given in Table 1. The timely distance between surgeries can be taken from S3 Table.

**Table 1:** Number of presurgical hand preparations performed in phase 1 and 2 of the study.

Number of presurgical preparations conducted	Phase 1 “routine practice protocol”			Phase 2 “standardised protocol”		
	Surgeons (n = 2^a^)	Staff (n = 4^b^)	Students (n = 16^c^)	Surgeons (n = 2^a^)	Staff (n = 5)	Students (n = 16^c^)
	20	10	16	20	5	16
**Total number per study phase**	46			41		

^a^Each surgeon prepared for ten surgical interventions accounting for a total of 20 surgeries in phase 1 and 2, respectively. Time between surgeries and hand asepsis procedures was at least one day except for surgeon B who once had two surgeries at the same day (S3 Table).

^b^Three staff members participated in four, three and two surgical interventions, respectively (S3 Table).

^c^Students participated only once (S3 Table).

### Phase 2 –hand preparation according to a standardised protocol

In phase 2, a standardised protocol for presurgical hand disinfection was implemented. The protocol was deduced from the “VAH method 12” outlined in the “Requirements and methods for certification of chemical disinfection procedures” given by the German Association for Applied Hygiene (VAH) Disinfectants Commission [34] which is one of the most important German reference institutions for issuance of certificates and listing of disinfection procedures. “VAH method 12” is based on DIN EN 12791:2018–01 (Chemical disinfectants and antiseptics—Surgical hand disinfection—Test method and requirements (phase 2, step 2); German version). A contact time of 3 min was chosen based on the VAH reference method for surgical hand disinfection, i.e. 60vol% Propan-1-ol for 3 min. Detailed practical guidance on the different steps of surgical hand preparation was given to all participants by the lead author. In addition, instructions with clear illustrations were posted next to the washbasins.

The standardised protocol was as follows: (1) Wash with Lifosan^®^ soft (B. Braun) without brushing for 1 min. (2) Dry hands using a sterile towel. (3) Start rub-in procedure for a contact time of 3 min. Apply disinfectant to the cupped hand. Rub the hands using the following order: palm to palm including wrists, right palm over left dorsum with interlaced fingers and vice versa, palm to palm with fingers interlaced and backs of fingers to opposing palms, rotational rubbing of left thumb clasped in right palm and vice versa, rotational rubbing backwards and forwards with clasped fingers of right hand in left palm and vice versa [34, 35]. (4) Repeat every step five times to complete one cycle. (5) Repeat cycles continuously whilst keeping hands wet over the period of 3 min [34]. In preceding tests, the total amount of disinfectant needed to keep hands wet for 3 min was measured and was 12 ml (glove sizes S, M) and 18 ml (glove sizes L, XL) of disinfectant, respectively. Disinfection of the forearms (by rubbing forearm against forearm) was performed additionally for surgical interventions operated deeply within the abdomen. Hands were gloved after complete evaporation of Sterillium^®^. Each surgeon prepared for ten surgical interventions in study phase 2. Staff members and students participated only once. The timely distance between surgeries can be taken from S3 Table. The number of presurgical preparations conducted in phase 2 is also given in Table 1.

### Statistical analyses

Prior to sampling, the sample size was calculated using G*Power (a priori type statistical power analysis; two-tailed, α = 0.01, power 0.8, effect size 0.85) [36, 37]. The results obtained during phase 1 and 2 of the study were entered and prepared in Microsoft Excel. Percentage reductions were calculated from the bacterial counts before and after hand washing, and after disinfection. In addition, mean log_10_-reductions (LR) were calculated for the difference between after hand washing and after disinfection.

Differences in the relative bacterial count reduction of phases 1 and 2 were investigated using a Mann-Whitney-Test. In this context, the differences from before to after hand washing as well as the differences from after hand washing to after hand disinfection were considered. Since one aim of this study was to detect a significant improvement in the relative bacterial count reduction after disinfection by using a standardised protocol, the significance level was set to 0.01 in this analysis. The percentage increase was calculated from the bacterial counts obtained after hand disinfection and after surgery.

The relative increase in bacterial counts as a function of the duration of surgical procedures and glove perforations was investigated using ANOVA with a defined p-value of 0.05. Only those samplings with a valid result obtained after disinfection and after surgery were included in statistical analyses. Moreover, outliers (i.e. values which lay far beyond the majority of the other values) were eliminated before running the ANOVA [38]. This was necessary as most parametric statistics are not very robust against outliers which may decrease statistical power. All statistical analyses were done using SPSS Statistics 26 (IBM Deutschland GmbH, Ehningen, Germany).

## Results

### Surgical hand disinfection

#### Hand preparation habits observed in phase 1

**Fig 1:**
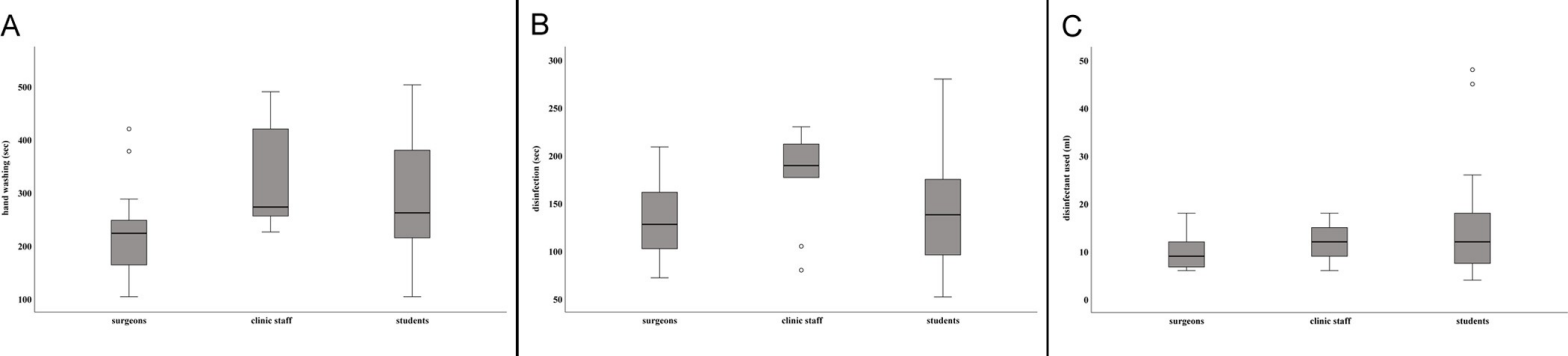
Presurgical hand preparation observed during phase 1 of the study. (A) Duration of washing hands (sec). (B) Duration of hand disinfection (sec). (C) Amount (ml) of disinfectant used. Box plots demonstrate minimum, first quartile, median, third quartile and maximum amount. The horizontal line within each box represents the median for duration of hand washing and disinfection (sec), and the median amount of disinfectant used, respectively; error bars indicate minimum and maximum. Open circles indicate values that are 1.5–3 times outside the interquartile range from the first or third quartile. Results represent data from 46 presurgical preparations collected at 20 surgical interventions.

Mean duration of hand washing among surgeons was 229 sec (± 76 sec) compared to 328 sec (± 97 sec) among clinic staff, and 273 sec (± 112 sec) among students (Fig 1A). Details about personal hand preparation habits are summarised in Table 2. The frequency of soaping and rinsing was six times at maximum. Disinfection was performed over 133 sec on average (± 39 sec) among surgeons, 179 sec (± 49 sec) among clinic staff, and 147 sec (± 62 sec) among students (Fig 1B). The amount of disinfectant used varied between 4 ml and 48 ml (Fig 1C). Fig 1A–1C distinctly shows that there was a large variation in performing surgical hand disinfection. Overall, the largest differences were found among students, although it should be mentioned that they also formed the largest group of participants. However, even for surgeons a wide variation in the duration of hand washing and hand disinfection was noticed. Mean bacterial counts obtained from each participant in phase 1 are given in S3 Table.

**Table 2:** Preparation of surgical hand asepsis observed in phase 1 of the study.

Observation	Documented cases during phase 1		
	Surgeons	Staff	Students
Usage of nail cleaners	0	0	0
Usage of a sponge	12	6	14
Usage of a brush	19	10	16
Nails only	3	0	6
Nails and/or hands	16	10	10
Forearms additionally	12	7	5
Soaping and rinsing 1x	6	0	3
Soaping and rinsing 2-5x	14	10	12
Soaping and rinsing >5x	0	0	1

It was observed that the distribution of the disinfectant was mostly irregular with thumbs, fingertips and backside of the fingers most frequently forgotten. None of the participants used a consistent or standardised rubbing technique.

#### Efficacy of presurgical hand preparation

**Fig 2:**
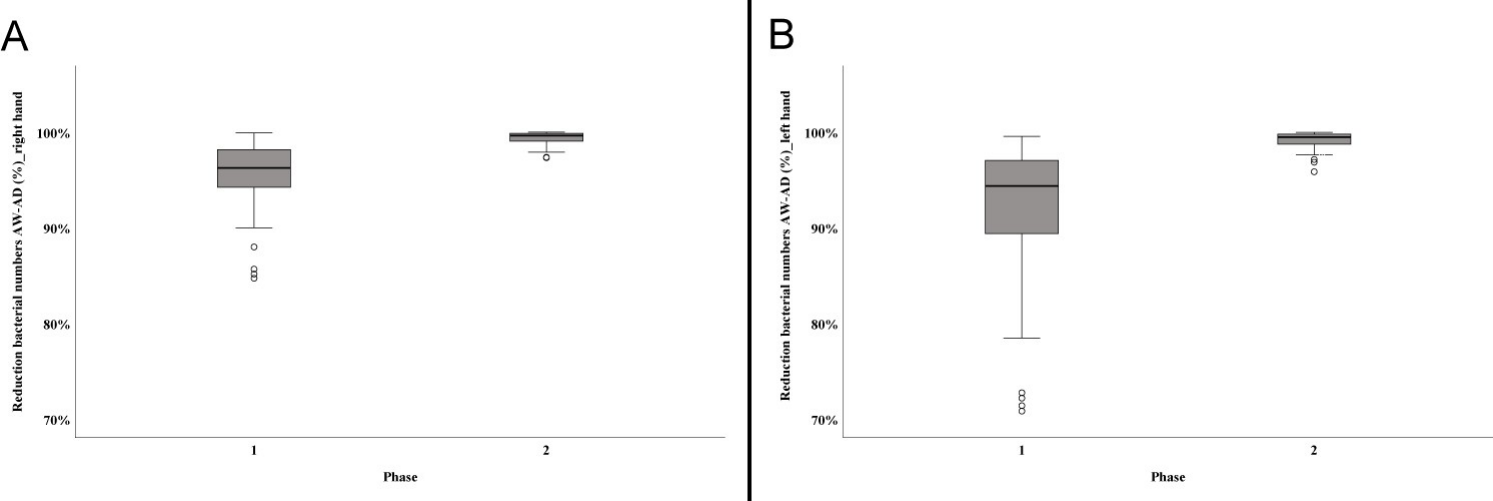
Efficacy of disinfection using two different protocols. The figure illustrates the reduction of bacterial numbers in %. (A) Decrease in bacterial numbers on the right hand determined in phase 1 and phase 2 of the study. (B) Decrease in bacterial numbers on the left hand determined in phase 1 and phase 2 of the study. Box plots demonstrate minimum, first quartile, median, third quartile and maximum amount. The horizontal line within each box represents the median; error bars indicate minimum and maximum. The open circles indicate values that are 1.5–3 times the interquartile range from the first quartile. Data were obtained from a total of 46 (phase 1) and 41 (phase 2) presurgical preparations, respectively. AW—after hand washing; AD—after disinfection.

Mean bacterial numbers obtained from each participant at each step of sampling are given in S3 Table. Time for hand washing had no significant influence on germ reduction as bacterial counts determined after washing did not differ significantly between phase 1 (individual routine practice with up to 8 min of hand washing) and phase 2 (standardised protocol with 1 min hand wash) (right hand p = 0.270, left hand p = 0.031). In contrast, the decrease in bacterial numbers seen after disinfection using the standardised protocol was highly significant (right hand p<0.001, left hand p<0.001). For the right hand, the mean rank in phase 1 was 28.44 and 60.44 in phase 2. For the left hand, a similar result was shown with a mean rank of 26.11 for phase 1 and 62.59 for phase 2. The strength of the effect according to Cohen (1992) [39] is r = 0.63 (right hand) and r = 0.73 (left hand) and thus corresponds to a strong effect. The mean reduction in phase 1 was 90.72% (LR = 3.23; right hand) and 89.97% (LR = 3.28; left hand) in contrast to a mean reduction of 98.85% (LR = 3.29; right hand) and 98.92% (LR = 3.47; left hand) in phase 2 (Fig 2).

#### Bacterial numbers determined after surgery

**Fig 3:**
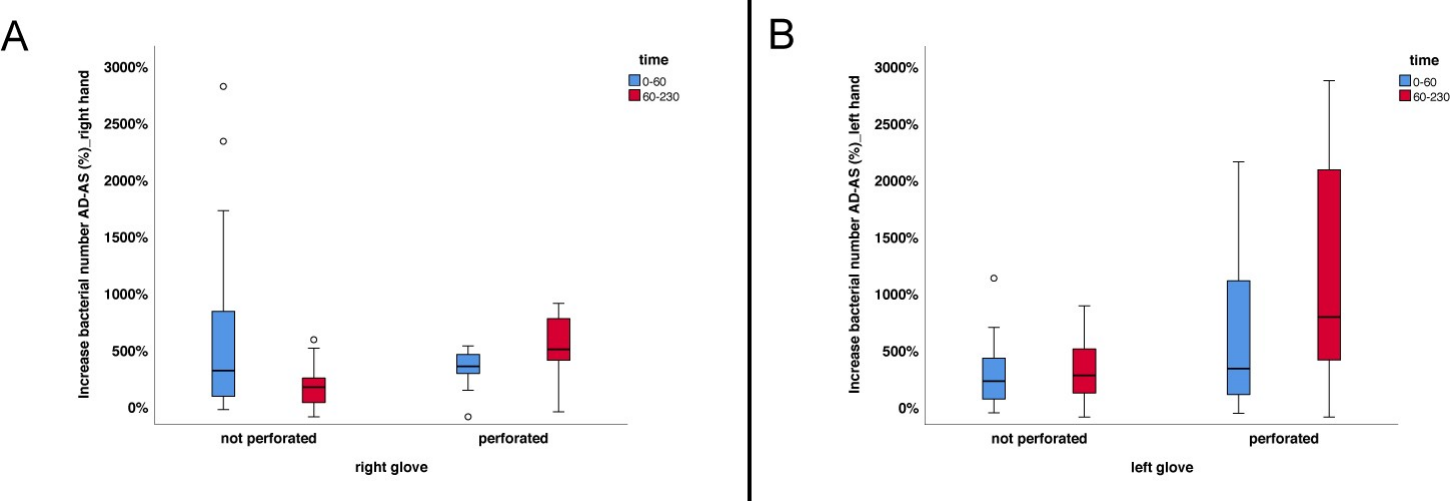
Increase in bacterial numbers determined after surgery. The figure shows the shift in bacterial counts with regard to glove perforation and duration of the surgical intervention. (A) Results obtained for the right hand. (B) Results obtained for the left hand. Box plots demonstrate minimum, first quartile, median, third quartile and maximum amount. The horizontal line within each box represents the median; error bars indicate minimum and maximum. The circles indicate values that are 1.5–3 times the interquartile range from the first or third quartile. Data were obtained from a total of 72 presurgical preparations. AD—after disinfection; AS—after surgery.

Independent of the study phase, an increase in bacterial numbers was mainly noticed after surgery (Fig 3A and 3B). In order to investigate, whether the time for surgery, the perforation of gloves, or both has an influence on bacterial numbers, 72 data sets each for the left and the right hand could be evaluated statistically. For the right hand, neither the investigated effect of time (p = 0.603) nor perforation of the glove (p = 0.741) proved to be significant (Table 3). The interaction of both effects significantly influenced bacterial numbers (p = 0.036) (Table 3). In contrast, the effect of a perforated glove (p<0.001), the duration of the surgery (p = 0.003), and the interaction between perforated glove and duration of the surgery (p = 0.027) were found to be significant for the left hand (Table 3). The entire model investigated using ANOVA proved to be significant with p<0.001.

**Table 3:** Results of ANOVA for between-subjects effects.

Source	Left hand					Right hand				
	Type III sum of squares	df	Mean square	F	Sig.	Type III sum of squares	df	Mean square	F	Sig.
Corrected model	8973980.041^a§^	3	2991326.680	10.730	<0.001	1590559.240^a#^	3	530186.413	2.034	0.117
Intercept	25826427.151	1	25826427.151	92.638	<0.001	9304583.921	1	9304583.921	35.697	<0.001
Glove perforated	6887662.671	1	6887662.671	24.706	<0.001	28801.573	1	28801.573	0.110	0.741
Time	2602003.249	1	2602003.249	9.333	0.003	71204.176	1	71204.176	0.273	0.603
Glove perforated * time	1430826.861	1	1430826.861	5.132	0.027	1194054.100	1	1194054.100	4.581	0.036
Error	18957558.350	68	278787.623			17724619.438	68	260656.168		
Total	47288804.891	72				33932071.302	72			
Corrected total	27931538.391	71				19315178.677	71			

Only those samplings with a valid result obtained after disinfection and after surgery were included in statistical analyses. Seventy-two gloves of the left hand (28 perforated, 44 intact) and 72 gloves of the right hand (22 perforated, 50 intact) could be included.

a^§^ –R Squared = 0.321 (adjusted R Squared = 0.291);

a#–R Squared = 0.082 (adjusted R Squared = 0.042); df–degrees of freedom; F–value of F-statistic; Sig.–significance

### Bacteria found on hands

Grampositive as well as gramnegative bacteria were isolated from hands. The grampositive bacteria were *S*. *aureus*, *S*. *caprae*, *S*. *chromogenes*, *S*. *equorum*, *S*. *haemolyticus*, *S*. *lugdunensis*, *S*. *pasteuri*, *S*. *pseudintermedius*, *S*. *saprophyticus*, *S*. *warneri* and *S*. *xylosus*, *Streptococcus* (*Sc*.) *equi* subsp. *zooepidemicus*, *Sc*. *oralis*, *Sc*. *pluranimalium*, and *Sc*. *sanguinis*. Gramnegative bacteria included three species of *Enterobacteriaceae*: *Citrobacter koseri*, *Escherichia coli*, and *Klebsiella oxytoca*. *Citrobacter koseri* was isolated from the left hand of surgeon A in a total of fifteen presurgical preparations at all steps of sampling. It was found out that the skin of the surgeon’s left palm was dry and cracked. Retrospective analysis of data obtained during phase 1 of the study revealed that surgeon A usually brushed nails, palms and rear surfaces of his hands.

*S*. *aureus* (n = 17) was cultured from a total of eleven participants (nine students, one staff member, one surgeon). Of the seventeen *S*. *aureus*, fourteen were characterised as MRSA and originated from eight of the 42 study participants (19%); seven students and one surgeon. Seven of the MRSA isolates were obtained from surgeon B (n = 2 in phase 1; n = 5 in phase 2); from each of others, MRSA was isolated only once. Surgeon B had contact to patients before entering the surgery room or used one of the computers at the clinic (Tables 4 and S1). All students carrying MRSA also reported having contact to patients before. Before hand-washing, samples of seven participants revealed MRSA in study phase 1. In four of these, MRSA was still present after disinfection in phase 1. In contrast, MRSA was grown from only one sample taken after surgery (phase 1). In phase 2, MRSA were detected before (6 participants) and after washing hands (2 participants), and after surgery (2 participants) but not in any sample taken after disinfection. All MRSA were allocated to *spa* type t011 or t6575 within the clonal complex CC398. With the exception of *spa* type t6575 all isolates were additionally grouped within the horse-specific subpopulation of CC398 (t011, ST398, gentamicin-resistant). Characteristics of all isolates are given in Table 4.

**Table 4:** Characterisation of MRSA isolates.

Isolate No.	*spa*-Type	Clonal complex	Resistance phenotype
33^a^	t011	CC398	PEN, OXA, GEN, TET, CIP, MOX, CEF, OXA/SU
37^a^	t011	CC398	PEN, OXA, GEN, TET, CIP, MOX, CEF, OXA/SU
38^a^	t6575	CC398	PEN, OXA, ERY, CLI, TET, CEF, OXA/SU
45^b^	t011	CC398	PEN, OXA, GEN, TET, CIP, MOX, CEF, OXA/SU
47^a^	t011	CC398	PEN, OXA, GEN, TET, CIP, MOX, CEF, OXA/SU
48^a^	t011	CC398	PEN, OXA, GEN, TET, CIP, MOX, CEF, OXA/SU
69^a^	t011	CC398	PEN, OXA, GEN, TET, CIP, MOX, CEF, OXA/SU
70^a^	t011	CC398	PEN, OXA, GEN, TET, CIP, MOX, CEF, OXA/SU
71^a^	t011	CC398	PEN, OXA, GEN, TET, CIP, MOX, CEF, OXA/SU
83^a^	t011	CC398	PEN, OXA, GEN, TET, CIP, MOX, CEF, OXA/SU
89^a^	t011	CC398	PEN, OXA, GEN, TET, CIP, MOX, CEF, OXA/SU
91^a^	t011	CC398	PEN, OXA, GEN, TET, CIP, MOX, CEF, OXA/SU
94^b^	t011	CC398	PEN, OXA, GEN, TET, CIP, MOX, CEF, OXA/SU
95^a^	t011	CC398	PEN, OXA, GEN, TET, CIP, MOX, CEF, OXA/SU

^a^Contact to horses.

^b^Working on the computer.

CIP, ciprofloxacin; CLI, clindamycin; ERY, erythromycin; CEF, cefoxitin; GEN, gentamicin; MOX, moxifloxacin; OXA, oxacillin; OXA/SU, oxacillin/sulbactam; PEN, penicillin; TET, tetracycline.

### Glove perforation

Over the study period, a total of 174 gloves were collected and tested for perforation (Table 5). The mean duration of a surgery was 76 ± 45 min. Invasive procedures included soft tissue and orthopaedic procedures (excluding arthroscopies). Minimally invasive procedures were arthroscopies. The occurrence of glove perforations was higher for surgeons (54%) compared to assistants (17%). In 85% of surgical interventions monitored, glove perforation was unnoticed by the participants. Perforations occurred most frequently in both gloves (Table 5) with thumb and index finger mostly affected. The occurrence of glove perforation for invasive procedures was 39% (52/134) compared to 18% (7/40) for minimally invasive procedures. A higher proportion of perforations (41% of gloves) occurred during surgical procedures with a duration of >60 min, compared to procedures ≤60 min (29% of gloves ruptured).

**Table 5:** Incidence of glove perforations noticed after surgery.

Variable	Number of gloves per variable (total number = 174)	Number of gloves perforated			
		total (%)	both gloves (%)	left glove only (%)	right glove only (%)
**Surgery type**					
Invasive	**134**	**52/134 (39%)**	**34/52 (65%)**	**14/52 (27%)**	**4/52 (8%)**
Soft tissue	122	46/122 (38%)	30/46 (65%)	12/46 (26%)	4/46 (9%)
Orthopaedic	12	6/12 (50%)	4/6 (67%)	2/6 (33%)	0/6 (0%)
Minimally invasive (Arthroscopy)	**40**	**7/40 (18%)**	**4/7 (57%)**	**0/7 (0%)**	**3/7 (43%)**
**Total**	**174**	**59/174 (34%)**	**38/59 (64%)**	**14/59 (24%)**	**7/59 (12%)**
**Surgery duration**					
≤60 min	98	28/98 (29%)	16/28 (57%)	9/28 (32%)	3/28 (11%)
>60 min	76	31/76 (41%)	22/31 (71%)	5/31 (16%)	4/31 (13%)
**Participant**					
Surgeon	80	43/80 (54%)	28/43 (65%)	11/43 (26%)	4/43 (9%)
Assistant	94	16/94 (17%)	10/16 (63%)	3/16 (19%)	3/16 (19%)

## Discussion

The two aspects of this study were 1) to assess personal habits for presurgical hand preparation among personnel and veterinarians-in-training at an equine hospital, and 2) to compare the efficacy of hand asepsis done as routine every-day practice (phase 1) versus a standardised protocol (phase 2) in reducing bacteria from hands. The prevalence of the horse-specific MRSA subpopulation of CC398 (t011, ST398, gentamicin-resistant) was determined, additionally. Although there are guidelines on hand hygiene given by the WHO [26], analysis of phase 1 revealed that presurgical preparation greatly varied among participants. This is in accordance to international surveys among veterinary surgical specialists which revealed that approximately 70% of the participants did not adhere to state-of-the-art protocols [3, 4]. Overall, the highest variation was found among students, but it should be noticed that they also formed the largest group of participants. Most of our participants mainly focused on hand washing (up to 8 min) whereas the mean application time of disinfectant was less than 3 min. The majority brushed nails, hands and forearms, though excessive washing or brushing does not increase efficacy and the use of presurgical hand-rubbing should be favoured over scrubbing [26, 40]. However, it is conceivable that a brush may be beneficial on visibly dirty hands before entering the surgery room not only in large animal clinics [26]. Excessive brushing is widely known to damage skin thereby increasing the risk of colonisation by pathogenic bacteria [41]. Moreover, the application of alcoholic disinfectants on damaged skin may lead to unpleasant burning and skin irritation resulting in less compliance [5]. As has been described elsewhere, washing of hands can be shortened to one min because time for hand washing had no significant influence on germ reduction (p = 0.270 and p = 0.031), though hand washing with neutral soap can remove loosely adherent transient skin flora [5, 26]. Total bacterial counts obtained in both phases of our study were similar to other reports [21, 22, 42–47]. Nevertheless, initial bacterial counts might have been underestimated in our study, because in nearly half of the investigations, participants reported about hand washing and/or disinfection prior to sampling (S1 Table). In phase 1, the relative reduction in bacterial numbers after disinfection also varied widely which might be attributed to personal habits. Introducing a standardised protocol completely eliminated personal effects and resulted in a highly significant reduction in bacterial numbers in phase 2 (approximately 99%) compared to phase 1 (approximately 90–91%) of the study on both hands. Education and training are important and critical for improving hand hygiene practices [26]. At our faculty, students are trained in hand hygiene and hand asepsis based on the WHO guidelines at the beginning of their third year of study. Nevertheless, compliance to these guidelines might not consistently be successful and students need to be trained repeatedly for long-lasting improvement. Hence, a guiding standardised protocol, e.g. presented in the form of images, and a timer, are highly recommended particularly for trainees and students with little experience in presurgical procedures.

The method we used to obtain total colony counts was in accordance to others, however, although obligatory according to the VAH method we used and recommended by other investigators, neutralising agents were not added to the sampling fluid in our investigations [21, 25, 26, 42]. Adding neutralising agents directly into the sampling fluid, which is exposed to the hands, reduces bias towards false positive efficacy of the disinfectant [25]. On the other hand, growth-enhancing properties have been attributed to the neutralizer leading to higher bacterial numbers [25]. Moreover, when testing disinfectants for virucidal activity, no specific neutralizers are required as they might be harmful to the cell lines used for viral growth. During testing, the virucidal activity must be immediately suppressed after contact-time by dilution in ice-cold diluent which is defined as cell culture medium supplemented with foetal calf serum [48]. As our study was performed in a veterinary clinic, we decided to abstain from adding a neutralizer because the residual effect of the disinfectant is desired in surgical hand asepsis [25]. This might have been resulted in an overestimation of disinfectant efficiency and can be seen as a major limitation of our study. However, we used only Sterillium^®^ and sampling as well as sample processing was performed in a consistent manner. Therefore, the bias introduced would be identical for all results.

A wide range of bacteria was isolated including human- and animal-associated coagulase-negative and -positive staphylococci [49]. Of bacteria which have been associated with SSI in horses, *S*. *aureus*, MRSA, *S*. *lugdunensis*, *S*. *pseudintermedius*, *Sc*. *equi* ssp. *zooepidemicus*, *Escherichia coli*, and *Klebsiella oxytoca* were found [28–32]. Eleven of 42 participants (26.2%) harboured *S*. *aureus* and 19.0% (8/42) carried MRSA on their hands which is more than the reported prevalence of MRSA colonisation in equine veterinarians [18, 50]. The majority of MRSA strains was allocated to the *spa* type t011 within the clonal complex CC398. MRSA CC398 is well established as a nosocomial pathogen in veterinary clinics and nasal colonisation of staff attending horses has been reported [15, 18]. The *spa* type t011-isolates could further be assigned to a subpopulation which is particularly associated with equine clinics [15, 18]. The risk of mutual transmission between horses and humans and the need for surveillance at the equine-human interface has been recently described [18]. The fact that mainly students carried MRSA highlights the necessity to raise awareness for identifying MRSA-colonised patients and environmental reservoirs, transmission routes, barrier precautions, and hand hygiene. This is strengthened by the finding that samples from one surgeon were repeatedly MRSA-positive. Among the potential reasons for that are: nasal carriage of MRSA by this participant, MRSA-carriage of patients which were handled immediately before sampling, contamination of equipment or surfaces at the premises serving as a reservoir. Nasal carriage of MRSA by horses and staff could not be investigated during this study but is part of an ongoing survey to be published elsewhere. MRSA challenges infection prevention and control interventions in equine healthcare and the utility of hand hygiene for prevention of MRSA colonisation in veterinary personnel has been demonstrated [51, 52]. Our data set is not large enough to support a statistical analysis. However, MRSA was mainly reduced by disinfection at least below the level of detection which underlines the findings that alcoholic hand rubs have an impact on MRSA [53]. The variety of other pathogenic and also zoonotic bacteria isolated at different steps of sampling underlines the need for proper hand hygiene not only when preparing for surgery. Moreover, the repeated isolation of *Citrobacter koseri* from the left hand of surgeon A shows that skin care is crucial to maintain the natural skin barrier and to prevent colonisation with transient pathogenic bacteria.

Glove perforation most often occurred in gloves worn by surgeons and in gloves used in invasive procedures. This was in accordance to other investigations in small and large animal surgery which suggested that invasive procedures and surgeons are associated with a higher risk of glove perforation [19, 20, 54–56]. Differences in glove perforation rates associated with the material of the gloves (i.e. latex gloves versus latex-free gloves) were not evaluated. Glove perforation (p<0.001), time (p = 0.003), as well as the interaction between both, glove perforation and time of surgery (p = 0.027), resulted in a significant relative increase in bacterial counts on the left hand. Contrary to this, only the combination of glove perforation and time significantly influenced bacterial numbers on the right hand. A possible explanation might be that perforations more frequently occur in gloves on the nondominant (mainly the left) hand which are punctured by suture needles or instruments held in the dominant hand [20, 56, 57]. Moreover, the nondominant hand is rather used for gross manipulations [57]. Furthermore, glove perforations increase with the duration of glove wear [58]. The modified WLT used enables to determine the location of any leak but might underestimate the number of detected perforations [19, 20, 57]. In accordance to other studies, surgical interventions of ≤60 min resulted in less glove perforations compared to procedures of >60 min [19, 20, 56]. Glove perforations have been reported to increase the risk for SSI in human surgery [19, 20]. However, the association of glove perforation and SSI was not examined in this study, in part because we became aware of only one horse that developed SSI.

Hand asepsis is an important and simple hygiene measure to decrease the risk of nosocomial infections. To the authors best knowledge, the aspect of comparing “general practice” in presurgical hand preparation to a standardised protocol has not been investigated so far. Our results demonstrate that guiding standardised protocols highly improve the efficacy of hand hygiene measures thereby ensuring compliance with established protocols.

## Supporting information

S1 Table Activities prior to surgery reported by the study participants. Prior to each presurgical preparation, detailed information was queried using a standardised in-house questionnaire with main focus on the participants’ previous activities on that day. The table summarises results from 87 questionnaires.
(DOCX)

S2 Table Bacterial numbers (cfu/ml) obtained in a laboratory test series using the standardised hand disinfection protocol and two different sampling fluids. After hand disinfection for 3 min according to the standardised protocol, hands were immersed in 100 ml sterile phosphate buffered saline (pH 7.2) or neutralizer EIII filled into polypropylene bags while holding the bag tightly around the participant´s wrist. The fluid was thoroughly dispersed all-over the surfaces of each hand for 1 min. EIII was composed of tryptic soy broth with 0.3% lecithin, 0.1% L-histidine, 0.5% sodium thiosulfate, and 3% Tween 80. Samples were used undiluted and in a dilution of 1:10. A 100 μl-aliquot of each undiluted and serially diluted sample was inoculated onto Columbia agar plates in duplicates using sterile spreaders. After 48 h of incubation at 37°C, bacterial colonies were manually counted and mean colony-forming units (cfu)/ml were calculated.
(DOCX)

S3 Table Data collected at a total of 87 presurgical preparations using Sterillium^®^ for surgical hand disinfection. Surgeons A and B prepared for 10 surgical interventions per study phase. In phase 1, three staff members (staff 2, 3, and 4) participated in four, three and two surgical interventions, respectively. Students participated only once. The timely distance given does not necessarily mean that the respective surgeon had no surgery in-between. Mean bacterial counts given were determined from colonies counted on agar plates inoculated in duplicates. BW–before washing; AW–after washing; AD–after disinfection; AS–after surgery; ne–not evaluable; na–not applicable, as this participant assisted in only one surgery in this phase of the study; a–time between surgery no. 10 of study phase 1 and surgery no. 1 in phase 2.
(DOCX)
