# Supplementary material for: Surgical hand preparation in an equine hospital: Comparison of general practice with a standardised protocol and characterisation of the methicillin-resistant Staphylococcus aureus recovered
Source: PLoS One. 2020 Dec 22;15(12):e0242961. doi: 10.1371/journal.pone.0242961 (PMC7755178; doi:10.1371/journal.pone.0242961)
Supplement: S1 Table — Prior to each presurgical preparation, detailed information was queried using a standardised in-house questionnaire with main focus on the participants’ previous activities on that day. The table summarises results from 87 questionnaires. (DOCX) [file pone.0242961.s001.docx]

**S1 Table. Activities prior to surgery as reported by the participants taken from a total of 87 questionnaires.**

| **Activity prior to presurgical hand asepsis** | **Times reported by** | | |
| --- | --- | --- | --- |
|  | **surgeons** | **staff** | **students** |
| Office work | 9 | 1 | 0 |
| Working at the computer | 13 | 1 | 1 |
| Being on the phone | 4 | 0 | 0 |
| Contact to horses (examination, treatment, assistance) | 11 | 10 | 27 |
| Washing hands after contact to horses | 3 | 2 | 9 |
| Touching surfaces in the hospital/stable, equipment | 10 | 2 | 7 |
| Hand wash ≤10 min ago | 1 | 2 | 6 |
| Hand wash >10 min ago | 1 | 4 | 8 |
| Hand wash (can’t remember how long ago) | 4 | 2 | 5 |
| Hand wash & disinfection | 0 | 0 | 4 |
| Hand disinfection | 0 | 0 | 4 |
