# Supplementary material for: Surgical hand preparation in an equine hospital: Comparison of general practice with a standardised protocol and characterisation of the methicillin-resistant Staphylococcus aureus recovered
Source: PLoS One. 2020 Dec 22;15(12):e0242961. doi: 10.1371/journal.pone.0242961 (PMC7755178; doi:10.1371/journal.pone.0242961)
Supplement: S2 Table — After hand disinfection for 3 min according to the standardised protocol, hands were immersed in 100 ml sterile phosphate buffered saline (pH 7.2) or neutralizer EIII filled into polypropylene bags while holding the bag tightly around the participant´s wrist. The fluid was thoroughly dispersed all-over the surfaces of each hand for 1 min. EIII was composed of tryptic soy broth with 0.3% lecithin, 0.1% L-histidine, 0.5% sodium thiosulfate, and 3% Tween 80. Samples were used undiluted and in a dilution of 1:10. A 100 μl-aliquot of each undiluted and serially diluted sample was inoculated onto Columbia agar plates in duplicates using sterile spreaders. After 48 h of incubation at 37°C, bacterial colonies were manually counted and mean colony-forming units (cfu)/ml were calculated. (DOCX) [file pone.0242961.s002.docx]

**S2 Table. Bacterial numbers (cfu/ml) obtained in a laboratory test series using the standardised hand disinfection protocol and two different sampling fluids.**

| **Test No.** | **Colony-forming units/ml** | |
| --- | --- | --- |
|  | **phosphate buffered saline** | **neutralizer EIII** |
| 1 | 15 | 41 |
| 2 | 40 | 27 |
| 3 | 40 | 600 |
| 4 | 165 | 190 |
| 5 | 145 | 14 |
| 6 | 0 | 170 |
| 7 | 10 | 36 |
| 8 | 75 | 5 |
| 9 | 20 | 300 |
| 10 | 405 | 140 |
| 11 | 15 | 23 |
| 12 | 20 | 73 |

Using EIII sometimes resulted in much higher cfu/ml compared to PBS. This can be attributed to its neutralising effect by eliminating residual activity of the disinfectant. Moreover, the neutraliser has bacterial growth-enhancing properties as it contains TSB and lecithin. Adding neutralising agents directly into the sampling fluid, which is exposed to the hands, reduces further bias towards false positive efficacy (Biermann et al. 2019) [25]. As our study was performed in a veterinary clinic, we decided not to follow this protocol because the residual effect of the disinfectant is desired in surgical hand asepsis.
