# Supplementary material for: Surgical hand preparation in an equine hospital: Comparison of general practice with a standardised protocol and characterisation of the methicillin-resistant Staphylococcus aureus recovered
Source: PLoS One. 2020 Dec 22;15(12):e0242961. doi: 10.1371/journal.pone.0242961 (PMC7755178; doi:10.1371/journal.pone.0242961)
Supplement: S3 Table — Surgeons A and B prepared for 10 surgical interventions per study phase. In phase 1, three staff members (staff 2, 3, and 4) participated in four, three and two surgical interventions, respectively. Students participated only once. The timely distance given does not necessarily mean that the respective surgeon had no surgery in-between. Mean bacterial counts given were determined from colonies counted on agar plates inoculated in duplicates. BW–before washing; AW–after washing; AD–after disinfection; AS–after surgery; ne–not evaluable; na–not applicable, as this participant assisted in only one surgery in this phase of the study; a–time between surgery no. 10 of study phase 1 and surgery no. 1 in phase 2. (DOCX) [file pone.0242961.s003.docx]

**S3 Table. Data collected at a total of 87 presurgical preparations using Sterillium^®^ for surgical hand disinfection.**

| **Person no.** | **Study phase** | **Left hand** | | | | | **Right hand** | | | | | **Duration of surgery** | **Timely distance between surgeries** |
| --- | --- | --- | --- | --- | --- | --- | --- | --- | --- | --- | --- | --- | --- |
|  |  | **mean cfu/ml** | | | | **glove perforated (0 = no,**  **1 = yes)** | **mean cfu/ml** | | | | **glove perforated (0 = no,**  **1 = yes)** |  |  |
|  |  | **BW** | **AW** | **AD** | **AS** |  | **BW** | **AW** | **AD** | **AS** |  |  |  |
| Surgeon A | 1 | 37000 | 24000 | 1610 | 11600 | 0 | 25500 | 19300 | 143 | 867 | 1 | ≤60 min | na |
| Surgeon A | 1 | 31000 | 13800 | 550 | 1860 | 1 | 32000 | 6590 | 81 | 3050 | 1 | >60 min | 9 d |
| Surgeon A | 1 | 15300 | 6270 | 227 | 1050 | 1 | 6730 | 1270 | 10 | 143 | 0 | ≤60 min | 1 d |
| Surgeon A | 1 | 14100 | 5360 | 418 | 1480 | 0 | 5090 | 667 | 33 | 150 | 0 | >60 min | 1 d |
| Surgeon A | 1 | 15500 | 12600 | 1220 | 2860 | 1 | 14900 | 6500 | 36 | 4570 | 0 | ≤60 min | 3 d |
| Surgeon A | 1 | 21500 | 9320 | 600 | 2180 | 1 | 2000 | 1050 | 20 | 85 | 1 | >60 min | 1 d |
| Surgeon A | 1 | 41500 | 14500 | 714 | 286 | 1 | 40000 | 5550 | 96 | 364 | 1 | ≤60 min | 7 d |
| Surgeon A | 1 | 19500 | 3140 | 255 | 1640 | 1 | 6500 | 350 | 10 | 40 | 1 | >60 min | 1 d |
| Surgeon A | 1 | 64500 | 22000 | 714 | 5640 | 0 | 8500 | 6140 | 29 | 100 | 0 | ≤60 min | 13 d |
| Surgeon A | 1 | 10000 | 3670 | 41 | 4360 | 1 | 6500 | 524 | 20 | 182 | 1 | >60 min | 1 d |
| Surgeon B | 1 | 1140 | 164 | 6 | 91 | 1 | 857 | 633 | 20 | 76 | 1 | ≤60 min | na |
| Surgeon B | 1 | 22500 | 4000 | 355 | ne | 1 | 4680 | 524 | 15 | ne | 0 | >60 min | 49 d |
| Surgeon B | 1 | 7820 | 3270 | 473 | 1860 | 1 | 3910 | 667 | 96 | 600 | 0 | ≤60 min | 6 d |
| Surgeon B | 1 | 2320 | 714 | 20 | 450 | 1 | 6180 | 864 | 86 | 450 | 1 | ≤60 min | 15 d |
| Surgeon B | 1 | 1430 | 20 | 15 | 20 | 0 | 2910 | 143 | 157 | 364 | 0 | >60 min | 6 d |
| Surgeon B | 1 | 714 | 364 | 52 | 52 | 1 | 619 | 286 | 100 | 550 | 1 | ≤60 min | 22 d |
| Surgeon B | 1 | 7820 | 1570 | 181 | 2410 | 1 | 3090 | 429 | 20 | 25 | 0 | ≤60 min | 6 d |
| Surgeon B | 1 | 1860 | 250 | 24 | 591 | 1 | 1200 | 1000 | 120 | 2910 | 1 | >60 min | 16 d |
| Surgeon B | 1 | 9230 | 3410 | 71 | 429 | 0 | 6550 | 2230 | 157 | 455 | 0 | >60 min | 4 d |
| Surgeon B | 1 | 2860 | 1500 | 323 | 1860 | 1 | 4050 | 614 | 23 | 105 | 0 | ≤60 min | 3 h |
| Staff 1 | 1 | 2290 | 200 | 55 | 36 | 0 | 1400 | 300 | 16 | 139 | 0 | ≤60 min | na |
| Staff 2 | 1 | 55000 | 7820 | 245 | 2300 | 0 | 49000 | 2680 | 162 | 1100 | 0 | >60 min | na |
| Staff 2 | 1 | 12200 | 4910 | 864 | 3180 | 0 | 17600 | 12400 | 527 | 11100 | 0 | >60 min | 10 d |
| Staff 2 | 1 | 14900 | 4140 | 105 | 619 | 0 | 9730 | 3000 | 223 | 864 | 0 | >60 min | 12 d |
| Staff 2 | 1 | 19900 | 11700 | 314 | 2230 | 1 | 19900 | 2950 | 177 | 1640 | 1 | >60 min | 15 d |
| Staff 3 | 1 | 62000 | 3730 | 195 | 333 | 0 | 110000 | 7950 | 182 | 1100 | 0 | >60 min | na |
| Staff 3 | 1 | 13000 | 2190 | 76 | 1480 | 0 | 35500 | 5730 | 295 | 1680 | 1 | >60 min | 5 d |
| Staff 3 | 1 | 56000 | 14100 | 64 | 1380 | 1 | 37000 | 8820 | 73 | 2050 | 0 | >60 min | 22 d |
| Staff 4 | 1 | 22500 | 4000 | 355 | ne | 1 | 4680 | 524 | 15 | ne | 0 | >60 min | na |
| Staff 4 | 1 | 9230 | 3410 | 71 | 429 | 0 | 6550 | 2230 | 157 | 455 | 0 | >60 min | 75 d |
| Student 1 | 1 | 600 | 250 | 20 | 464 | 0 | 857 | 455 | ne | 30 | 0 | ≤60 min | na |
| Student 2 | 1 | 3180 | 238 | 18 | ne | 0 | 1550 | 476 | 73 | 40 | 0 | >60 min | na |
| Student 3 | 1 | 43600 | 13400 | 143 | 700 | 0 | 32600 | 15500 | 700 | 827 | 0 | >60 min | na |
| Student 4 | 1 | 13500 | 12800 | 3560 | 5820 | 0 | 32500 | 16000 | 3860 | 11500 | 0 | ≤60 min | na |
| Student 5 | 1 | 15700 | 4450 | 46 | 250 | 0 | 24000 | 4550 | 18 | 300 | 0 | ≤60 min | na |
| Student 6 | 1 | 6270 | 524 | 30 | 1910 | 0 | 5230 | 1300 | 52 | 952 | 0 | ≤60 min | na |
| Student 7 | 1 | 1550 | 200 | ne | ne | 1 | 1590 | 455 | 10 | 2000 | 1 | ≤60 min | na |
| Student 8 | 1 | 15700 | 3140 | 110 | 750 | 0 | 41500 | 4230 | 227 | 2860 | 0 | >60 min | na |
| Student 9 | 1 | ne | 2950 | 105 | 571 | 0 | ne | 3000 | 445 | 762 | 0 | ≤60 min | na |
| Student 10 | 1 | 2140 | 650 | 10 | 150 | 1 | 700 | 455 | 10 | 100 | 1 | >60 min | na |
| Student 11 | 1 | 295 | 120 | 35 | 190 | 0 | 450 | 273 | 15 | 20 | 0 | >60 min | na |
| Student 12 | 1 | 28300 | 26000 | 4340 | 6770 | 0 | 79500 | 33800 | 727 | 3770 | 0 | ≤60 min | na |
| Student 13 | 1 | 7000 | 1640 | 290 | 1180 | 0 | 9140 | 1650 | 371 | 1620 | 0 | ≤60 min | na |
| Student 14 | 1 | 2480 | 450 | 25 | 45 | 0 | 8950 | 4270 | 24 | 70 | 0 | >60 min | na |
| Student 15 | 1 | 15800 | 3140 | 30 | 100 | 0 | 9180 | 4500 | 4 | 1200 | 1 | ≤60 min | na |
| Student 16 | 1 | 667 | 100 | 29 | 350 | 0 | 3680 | 500 | 224 | 667 | 0 | ≤60 min | na |
| Surgeon A | 2 | 54500 | 51500 | 124 | 619 | 0 | 19200 | 17900 | 38 | 400 | 0 | ≤60 min | 41 d^a^ |
| Surgeon A | 2 | 79100 | 33300 | 436 | 823 | 0 | 27900 | 10100 | 64 | 332 | 0 | ≤60 min | 66 d |
| Surgeon A | 2 | 21800 | 12800 | 68 | 668 | 0 | 13300 | 6910 | 43 | 255 | 1 | >60 min | 4 d |
| Surgeon A | 2 | 46500 | 21100 | 227 | 405 | 0 | 26000 | 13000 | 100 | 219 | 0 | ≤60 min | 8 d |
| Surgeon A | 2 | 26500 | 17900 | 386 | 1150 | 0 | 10600 | 5450 | 110 | 323 | 0 | >60 min | 8 d |
| Surgeon A | 2 | 15100 | 14800 | 245 | 2160 | 1 | 6270 | 5860 | 55 | 322 | 1 | >60 min | 11 d |
| Surgeon A | 2 | 59300 | 33500 | 52 | 577 | 1 | 47800 | 11400 | 80 | 186 | 1 | ≤60 min | 8 d |
| Surgeon A | 2 | 17600 | 4770 | 114 | 3380 | 1 | 7360 | 2100 | 132 | 950 | 1 | >60 min | 1 d |
| Surgeon A | 2 | 7450 | 3500 | 145 | 514 | 1 | 6640 | 2680 | 43 | 368 | 0 | ≤60 min | 19 d |
| Surgeon A | 2 | 18000 | 12000 | 141 | 564 | 0 | 16500 | 13300 | 52 | 327 | 1 | ≤60 min | 2 d |
| Surgeon B | 2 | 1480 | 159 | 2 | 18 | 1 | 500 | 380 | 0 | 6 | 1 | ≤60 min | 22 d^a^ |
| Surgeon B | 2 | 13900 | 6180 | 30 | 10 | 1 | 3680 | 636 | 2 | 36 | 0 | ≤60 min | 21 d |
| Surgeon B | 2 | 2180 | 209 | 4 | 10 | 0 | 2360 | 205 | 2 | 0 | 1 | ≤60 min | 6 d |
| Surgeon B | 2 | 3590 | 500 | 3 | 14 | 0 | 5270 | 409 | 5 | 3 | 0 | ≤60 min | 9 d |
| Surgeon B | 2 | 10300 | 3180 | 7 | 146 | 1 | 2820 | 250 | 4 | 2 | 1 | >60 min | 12 d |
| Surgeon B | 2 | 1060 | 619 | 4 | 5 | 1 | 1410 | 355 | 2 | 2 | 0 | ≤60 min | 44 d |
| Surgeon B | 2 | 4860 | 160 | 3 | 3 | 0 | 3950 | 459 | 1 | 1 | 0 | ≤60 min | 6 d |
| Surgeon B | 2 | 7410 | 2410 | 1 | 2 | 1 | 6640 | 1590 | 1 | 8 | 0 | ≤60 min | 41 d |
| Surgeon B | 2 | 1030 | 476 | 2 | 2560 | 1 | 1160 | 500 | 1 | 891 | 0 | ≤60 min | 1 d |
| Surgeon B | 2 | 7180 | 3050 | 2 | 134 | 0 | 11100 | 667 | 2 | 50 | 0 | >60 min | 20 d |
| Staff 4 | 2 | 3590 | 500 | 3 | 14 | 0 | 5270 | 409 | 5 | 3 | 0 | ≤60 min | 58 d |
| Staff 5 | 2 | 54500 | 51500 | 124 | 619 | 0 | 19200 | 17900 | 38 | 400 | 0 | ≤60 min | na |
| Staff 6 | 2 | 60000 | 9050 | 659 | 641 | 0 | 15500 | 7050 | 264 | 450 | 0 | ≤60 min | na |
| Staff 7 | 2 | 2360 | 173 | 1 | 9 | 1 | 3580 | 541 | 0 | 1 | 1 | ≤60 min | na |
| Staff 8 | 2 | 116000 | 61400 | 73 | 3200 | 0 | 35000 | 15000 | 20 | 582 | 0 | ≤60 min | na |
| Student 17 | 2 | 5950 | 3820 | 77 | 195 | 0 | 2410 | 619 | 73 | 118 | 0 | >60 min | na |
| Student 18 | 2 | 16500 | 4140 | 50 | 4910 | 0 | 9450 | 4550 | 38 | 3270 | 0 | ≤60 min | na |
| Student 19 | 2 | 450 | 450 | 4 | 2 | 0 | 1270 | 591 | 2 | 8 | 0 | ≤60 min | na |
| Student 20 | 2 | 1000 | 620 | 3 | 4 | 0 | 8640 | 3140 | 5 | 10 | 0 | ≤60 min | na |
| Student 21 | 2 | 9550 | 4410 | 10 | 873 | 0 | 8180 | 382 | 10 | 82 | 1 | >60 min | na |
| Student 22 | 2 | 5680 | 1950 | 20 | 67 | 0 | 9770 | 4000 | 4.5 | 9 | 0 | ≤60 min | na |
| Student 23 | 2 | 2450 | 350 | 10 | 176 | 0 | 2330 | 476 | 10 | 100 | 0 | ≤60 min | na |
| Student 24 | 2 | 36500 | 7860 | 1 | 18 | 1 | 33800 | 7450 | 2 | 25 | 0 | ≤60 min | na |
| Student 25 | 2 | 11700 | 2000 | 1 | 2 | 0 | 8500 | 3090 | 1 | 4 | 0 | ≤60 min | na |
| Student 26 | 2 | 1640 | 1240 | 3 | 13 | 0 | 857 | 277 | 2 | 11 | 0 | ≤60 min | na |
| Student 27 | 2 | 42000 | 7140 | 27 | 71 | 1 | 16500 | 9080 | 9 | 40 | 1 | ≤60 min | na |
| Student 28 | 2 | 1550 | 955 | 30 | 90 | 0 | 1550 | 750 | 20 | 20 | 0 | ≤60 min | na |
| Student 29 | 2 | 3180 | 1910 | 1 | 35 | 0 | 3820 | 1760 | 2 | 68 | 0 | ≤60 min | na |
| Student 30 | 2 | 29200 | 23000 | 11 | 17 | 0 | 63300 | 28500 | 53 | 109 | 0 | ≤60 min | na |
| Student 31 | 2 | 1240 | 100 | 1 | 2 | 0 | 1090 | 100 | 1 | 2 | 0 | ≤60 min | na |
| Student 32 | 2 | 34100 | 14700 | 15 | 32 | 0 | 46900 | 21500 | 14 | 33 | 0 | >60 min | na |

Surgeons A and B prepared for 10 surgical interventions per study phase. In phase 1, three staff members (staff 2 – 4) participated in four, three and two surgical interventions, respectively. Students participated only once. Mean bacterial counts given were determined from colonies counted on agar plates inoculated in duplicates.

BW – before washing;

AW – after washing;

AD – after disinfection;

AS – after surgery;

ne – not evaluable;

na – not applicable, as this participant assisted in only one surgery in this phase of the study;

a – time between surgery no. 10 of study phase 1 and surgery no. 1 in phase 2
